# A Redox-Based Autoinduction Strategy to Facilitate Expression of 5xCys-Tagged Proteins for Electrobiofabrication

**DOI:** 10.3389/fmicb.2021.675729

**Published:** 2021-06-18

**Authors:** Sally Wang, Chen-Yu Tsao, Dana Motabar, Jinyang Li, Gregory F. Payne, William E. Bentley

**Affiliations:** ^1^Fischell Department of Bioengineering, University of Maryland, College Park, College Park, MD, United States; ^2^Fischell Institute for Biomedical Devices, University of Maryland, College Park, College Park, MD, United States; ^3^Institute of Bioscience and Biotechnology Research, University of Maryland, College Park, College Park, MD, United States

**Keywords:** cysteine tag, protein expression, inclusion body, autoinduction, biofabrication, redox

## Abstract

Biofabrication utilizes biological materials and biological means, or mimics thereof, for assembly. When interfaced with microelectronics, electrobiofabricated assemblies enable exquisite sensing and reporting capabilities. We recently demonstrated that thiolated polyethylene glycol (PEG-SH) could be oxidatively assembled into a thin disulfide crosslinked hydrogel at an electrode surface; with sufficient oxidation, extra sulfenic acid groups are made available for covalent, disulfide coupling to sulfhydryl groups of proteins or peptides. We intentionally introduced a polycysteine tag (5xCys-tag) consisting of five consecutive cysteine residues at the C-terminus of a *Streptococcal* protein G to enable its covalent coupling to an electroassembled PEG-SH film. We found, however, that its expression and purification from *E. coli* was difficult, owing to the extra cysteine residues. We developed a redox-based autoinduction methodology that greatly enhanced the yield, especially in the soluble fraction of *E. coli* extracts. The redox component involved the deletion of *oxyRS*, a global regulator of the oxidative stress response and the autoinduction component integrated a quorum sensing (QS) switch that keys the secreted QS autoinducer-2 to induction. Interestingly, both methods helped when independently employed and further, when used in combination (i.e., autodinduced *oxyRS* mutant) the results were best—we found the highest total yield and highest yield in the soluble fraction. We hypothesize that the production host was less prone to severe metabolic perturbations that might reduce yield or drive sequestration of the -tagged protein into inclusion bodies. We expect this methodology will be useful for the expression of many such Cys-tagged proteins, ultimately enabling a diverse array of functionalized devices.

## Introduction

Affinity tags incorporated into the primary sequences of recombinant proteins were initially developed as a means to facilitate their purification and/or detection (Terpe, 2003; Kimple et al., 2013). Because protein engineering is now fairly routine, the incorporation of “designer” tags has emerged enabling a variety of new functions, among those including protein attachment onto both biotic and abiotic materials. For example, a pentatyrosine pro-tag was shown to allow tyrosinase-mediated covalent coupling of an IgG-binding protein G or a human glycoprotein, ApoH, with both polysaccharides (Wu et al., 2009) and silk fibroin from *Bombyx mori* (Wu et al., 2020); a polyglutamine tag facilitated the assembly of proteins onto both gelatin (Liu et al., 2015) and spider silk (Wu et al., 2017); and a polylysine tag was added to enzymes for covalent tethering onto engineered tobacco mosaic virus-derived virus like particles (Bhokisham et al., 2020). Other peptide tags of varied amino acid composition enable binding onto solid materials such as gold (Tamerler et al., 2006; Adams et al., 2015; Terrell et al., 2021), silver (Sedlak et al., 2012), silicon (Zhou et al., 2015), as well as various hydrophobic surfaces (Tanaka et al., 2006) through non-covalent interactions. Methodologies that allow protein attachment to various substrates have created new possibilities to construct devices with diverse functions introduced by the assembled proteins. These “designer” proteins, however, can also present challenges in expression and purification, owing to the added tags (Kim et al., 2012; Lilie et al., 2013); yet their value is worth the challenge.

For example, we recently showed how a protein carrying a pentacysteine tag could be covalently tethered onto an electrode-assembled thiol-containing polyethylene glycol (PEG) hydrogel film. Film-associated thiol groups (Li et al., 2020) served as substrates for covalent assembly of engineered proteins, especially when electrochemically converted to sulfenic acid groups so that the cysteine-tagged proteins could rapidly and spontaneously form disulfide bonds. That is, the disulfide bonds were enabled by providing a redox mediator and an oxidizing voltage to the electrode so that the mediator abstracted electrons from the thiol, leaving the reactive sulfenic acid. In this way, the assembled proteins are restricted to the boundaries of the electrode, upon which the PEG is electroassembled. The same electrode surface can then serve as an electrochemical sensor with functionalized proteins to suit any purpose. Developing surfaces with functionalized PEG is attractive for many reasons, including detailed studies on a variety of biological interactions. For example, PEG is used as a mimic for extracellular matrix (ECM) (Zhang et al., 2008) and mucins found in epithelial tissues (Joyner et al., 2019); electrodeposited PEG could be functionalized with designer proteins and, because it is surface assembled, it also can be made accessible to various analytical measurements. As such, we showed how this film could be functionalized with a pentacysteine (5xCys)-tagged *Streptococcal* protein G to enable antibody-based immunoassays (Motabar et al., 2021). In the present study, however, we further show how the same 5xCys-tagged protein G, oxidatively assembled onto a PEG hydrogel can serve to capture cells onto an electrode surface, via protein G-assembled IgG.

Despite their versatility, the expression of cysteine-rich proteins has long been considered tricky in *E. coli* due to inherent issues brought about by the extra cysteine residues. Aggregation of cysteine-rich proteins usually results in inclusion body formation as the reduced state in the cytoplasm makes forming the disulfide bonds difficult (which enable proper folding). A variety of methods have been reported to tackle these issues, for instance, optimizing culture and purification conditions (Kiedzierska et al., 2008), recovering and re-folding active proteins from inclusion bodies (Singh et al., 2015), expression in the periplasm, and many others (Ban et al., 2020). In addition to this array of strategies, in this study we have coupled two, somewhat disjoint methodologies, but when combined lead to significantly increased yields of soluble protein.

First, we sought to reduce inclusion body formation with an autoinduction method mediated by rewiring the genetic circuitry of bacterial quorum sensing (QS) so that instead of mediating endogenous QS functions like biofilm formation, we engineered cells to induce expression of genes-of-interest. Overexpression of a recombinant protein is often accompanied by an imbalance of metabolism, often referred to as a “metabolic burden” (Bentley et al., 1990). In addition, the induction process is typically initiated by the addition of a bolus of inducer, which, in turn, can bring about significant transients in precursors or energy levels (Harcum and Bentley, 1993; Ramirez and Bentley, 1995; DeLisa et al., 2001a). This imposed stress could exhaust the bacterium’s innate protein folding and quality control machinery, leading to improperly folded proteins that aggregate and form inclusion bodies or get targeted for degradation. By rewiring the native quorum sensing system of *E. coli*, we had previously developed an autonomous induction system that used QS autoinducer signals (i.e., autoinducer 2, AI-2) to guide high level expression of recombinant proteins (Tsao et al., 2010).

**FIGURE 1:**
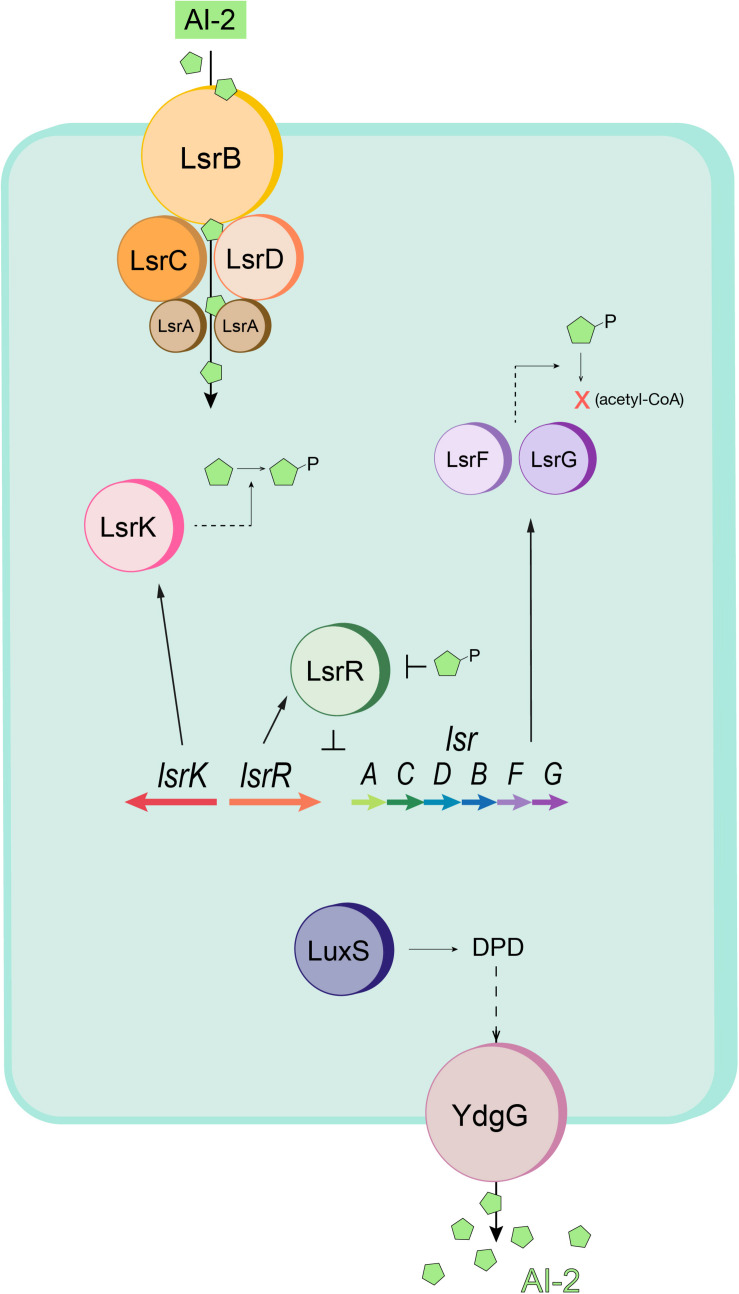
Schematic of the *E. coli* quorum sensing (Lsr) system. AI-2 (green pentagons) is imported into the cell by the Lsr transporter (LsrACDB) and is then phosphorylated to AI-2P by LsrK. When AI-2P binds LsrR and releases LsrR from the promoter region (thus modulating Lsr gene expression), AI-2 uptake is increased. Intracellular AI-2P is processed and degraded by LsrF and LsrG. LuxS produces DPD, the precursor to AI-2. The autoinducer is exported by YdgG (TqsA). Adapted from Wang S. et al. (2020).

As shown in Figure 1, *E. coli* naturally secrete AI-2 that accumulates in the culture fluids as the cells grow in number. When the autoinducer reaches a certain level, as indicated by reaching a “quorum” of cells, it is transported back inside via an ATP-dependent transporter where it is phosphorylated and initiates transcription from a LuxS-dependent regulon (Lsr) (Wang L. et al., 2005; Wang S. et al., 2020; Xavier and Bassler, 2005; Xavier et al., 2007). In Tsao et al., the native *lsr* promoter was used to induce the T7 polymerase that, in turn, was used to activate T7-based expression from a common pET vector. In essence, the added T7 circuit serves to amplify the original *lsr-*mediated expression. Aside from being a signal for cell-cell communication, AI-2 is produced by the enzymes Pfs and LuxS that are key elements in central metabolism (Vendeville et al., 2005), and because of this we reasoned that AI-2 might also serve as an indicator for the host’s metabolic state (DeLisa et al., 2001b). In fact, adding AI-2 by enhancing endogenous synthesis to cells overexpressing recombinant proteins served to increase yield (Tsao et al., 2011). Owing to the nature of QS, which allows an individual bacterium’s metabolic state to be probed and communicated to an entire population, we hypothesized that a QS-mediated autoinduction method would be “gentler” than the conventional, yet more “disruptive” IPTG induction method, and could cater to the expression and growth pace of the expression host.

Second, we sought to influence the ability of the cell to respond to oxidative/redox stresses, such as those that might accompany the rapid onset of protein synthesis where those proteins were potentially good substrates for sequestering reactive oxygen species (ROS). By deleting the oxidative stress regulon *oxyRS*, we not only attenuate the cell’s ability to respond to oxidative stress but hope to alter the reduced state of *E. coli*’s cytoplasm enabling a more oxidized state that favors disulfide bond formation. Several strains with deficiency in enzymes related to reduction of antioxidants (e.g., thioredoxin and glutathione) have shown to produce higher yields of properly oxidized proteins (Bessette et al., 1999; Faulkner et al., 2008). As a global transcription regulator, OxyR regulates the expression of many of these enzymes such as glutathione oxidoreductase (*gor*) (Becker-Hapak and Eisenstark, 1995) and alkyl hydroperoxide reductase (*ahpC*) (Storz et al., 1990; Seaver and Imlay, 2001). The non-coding RNA *oxyS*, too, is reported to regulate the oxidative state as in intracellular peroxide (H_2_O_2_) levels (Gonzalez-Flecha and Demple, 1999). Together, we aimed to investigate the effects of AI-2 mediated autoinduction and *oxyRS* deletion separately and in combination, to enable increased yield 5xCys-tagged protein G.

## Materials and Methods

### Bacterial Strains and Growth Media

The bacteria strains and plasmids used in this study are listed in Table 1. Luria-Bertani broth (LB) contained 5 g of yeast extract liter^–1^, 10 g of Bacto tryptone liter^–1^, and 10 g of NaCl liter^–1^ (Fisher). When necessary, media were supplemented with antibiotics at the following concentrations: ampicillin, 100 μg mL^–1^; kanamycin, 50 μg mL^–1^.

**TABLE 1:** Bacteria strains and plasmids used in this study.

	**Description**	**Source or references**
**Strain**		
NEB10β	Δ(*ara-leu) 7697 araD139 fhuA* Δ*lacX74 galK16 galE15 e14–*ϕ*80*d*lacZ*Δ*M15 recA1 relA1 endA1 nupG rpsL* (Str^*R*^)*rph spoT1* Δ(*mrr-hsdRMS-mcrBC*)	New England Biolabs
BL21 (DE3)	B strain, F^–^ *ompT hsdS*_*B*_ (r_*B*_^–^ m_*B*_^–^) *gal dcm rne131*λ(DE3)	Invitrogen
SW103	BL21 (DE3) Δ*oxyRS*, Cm^*R*^	This study
ZK126	*E. coli* K–12 substr. W3110 Δ*lacU169 tna-2*	Laboratory Stock
CT103	W3110 Δ*lsrFG*	This study
SW102	ZK126 Δ*oxyRS*	This study
**Plasmid**		
pET200/D-TOPO	Cloning vector, containing *T7* promoter, Km^*r*^	Invitrogen
pET-DsRed	pET200 derivative, containing *dsRed_Express_DR*	This study
pET-DsRedCys5	pET200 derivative, containing *dsRed_Express_DR* followed by 5xCys	Li et al., 2020
pET-G2Cys5	pET200 derivative, containing two copies of *Streptococcal* protein G Fc binding domain followed by 5xCys	Li et al., 2020
pCT5	pFZY1 derivative, containing *lsr* promoter fused with *T7RPol*, Ap^*r*^	Tsao et al., 2010
pET-E72G3	pET-32c derivative, containing three copies of *Streptococcal* protein G Fc binding domain	Tanaka et al., 2006

### Plasmid Construction

*Escherichia coli* strain NEB10β (New England Biolabs) was used for all subcloning. Proteins of interest (DsRed and *Streptococcus* protein G) were expressed with a C-terminal 5x cysteine tag (5x-Cys). Plasmid constructs are shown in Supplementary Figure 1. The sequence for the protein of interest was both preceded by six. His residues at the amino terminus and followed by five cysteine residues located at the carboxyl terminus. The coding sequence which comprised two copies of *Streptococcus* protein G’s Fc-binding domain (G2) and five Cys residues inserted at the carboxyl terminus was prepared by PCR amplification from a protein G template (pET-E72G3) (Tanaka et al., 2006; Wu et al., 2009) using primers listed in Supplementary Table 1. All oligonucleotides were purchased from Integrated DNA Technologies (Coralville, IA). The DsRed-5xCys fragment was prepared with similar procedures except with a DsRed template (pET-DsRed) and its corresponding primers (Supplementary Table 1). The PCR product was then purified through gel electrophoresis and inserted into a pET200 vector via the Champion^TM^ pET200 Directional TOPO^®^ Expression Kit (Invitrogen). The resultant plasmid that holds the protein of interest was confirmed by Sanger sequencing (Genewiz, NJ).

### Chromosomal Deletion of *oxyRS*, *lsrFG*

The one-step replacement method (Datsenko and Wanner, 2000) was used to construct deletions in *E. coli* BL21 (DE3), ZK126 and W3110. The phage λ Red recombination system was used to replace *oxyR, oxyS*, and the intergenic region with an *oxyRS*::*kan* PCR fragment. PCR was performed using pKD3 and pKD4 as a template, along with the primers oxyHP1 and oxyHP2 (Supplementary Table 1). The PCR products were treated with DpnI and introduced by electroporation into *E. coli* BL21 (DE3) and ZK126 containing plasmid pKD46, which expresses the λ Red recombinase, and was cured later by growth at 37°C. Recombinants were selected on LB supplemented with chloramphenicol or kanamycin. Deletions of *lsrF* and *lsrG* in *E. coli* W3110 were constructed similarly by PCR amplification of pKD3 with primers lsrHP3 and lsrHP2 (Supplementary Table 1). The deletion of genes in all cases was verified by PCR.

### Recombinant 5xCys-Tagged Protein Expression and Purification

For traditional IPTG-induced expression, overnight cultures of *E. coli* BL21 (DE3) (Invitrogen) and SW103 harboring plasmid pET-G2Cys5 in LB media were inoculated in 25 mL fresh media to OD_600_ = 0.10 in 250 mL flasks and incubated at 30 °C with shaking at 250 rpm. Upon reaching mid-log phase (OD_600_ ∼ 0.4–0.6), the cultures were induced with a final concentration of 1 mM IPTG (Sigma). For QS-mediated autoinduction, plasmids pCT5 (Tsao et al., 2010) and pET-G2Cys5 were electrically transformed into host strains CT103, SW102, and ZK126. All strains are *luxS*^+^ and synthesize AI-2 as a consequence of normal metabolism. Growth conditions were identical to that of BL21 (DE3) except no inducers were added. To purify 5xCys-tagged proteins, cells were harvested by centrifugation at 14,000 × g under 4 °C for 20 min. After lysis by BugBuster solution (Novagen) at room temperature for 40 min followed by sonication, the soluble cell extracts were mixed with Co^2+^ affinity resin (BD TALON^TM^, BD Biosciences), and the bound target proteins on the Co^2+^ were washed by phosphate buffer (pH = 7.4) (Sigma) three times to remove non-specifically bound proteins. The purified proteins were eluted with elution buffer (125 mM imidazole in phosphate buffer, pH = 7.4) for further experiments.

### Western Blot

Culture volumes equivalent to 2 mL at an OD_600_ of 1.0 were withdrawn from experiments 2, 4, and 6 h after IPTG induction of BL21 (DE3) cultures and centrifuged at 10,000 g for 10 min. The cell pellets were resuspended and lysed in 300 μL BugBuster protein extraction reagent (Novagen) at room temperature for 40 min and centrifuged again at 10,000 g for 10 min to separate soluble and insoluble cell fractions. A Bradford-based protein assay kit (Bio-Rad) was used to determine the protein concentration of the soluble fraction. Insoluble cell debris was resuspended with 0.1 mL resuspension buffer (0.1 M Phosphate Buffer [pH 6.8]). Both the soluble and insoluble fractions were mixed 1:1 (vol/vol) with sodium dodecyl sulfate (SDS) sample buffer [12.5% 0.5 M Tris-HCl (pH 6.8), 10% glycerol, 2% SDS, 5% β-mercaptoethanol, 0.0025% bromophenol blue], heated at 95°C for 10 min, and centrifuged at 4°C for 1 min. Samples with identical protein content, along with IMAC-purified protein G-5xCys (0.13 μg) were loaded onto 10% Mini-PROTEAN TGX Gels (Bio-Rad) for electrophoresis and blotted onto nitrocellulose membranes (Bio-Rad) using a Mini Trans-Blot cell (Bio-Rad) and Bjerrum Schafer-Nielsen transfer buffer (48 mM Tris, 29 mM glycine, 20% methanol) for 20 min at 15 V and 20 min at 20 V. The primary antibody, monoclonal antipolyhistidine (Sigma), was diluted 1:5,000 in antibody buffer [0.1% Tween 20 (vol/vol), Tris-buffered saline with 1% (wt/vol) nonfat dry milk] to probe protein G-5xCys. The membranes were then introduced to a solution of 1:50,000-diluted rabbit anti-mouse IgG conjugated with horseradish peroxidase (HRP) (Abcam). Membranes were developed using the Clarity Western ECL Substrate (Bio-Rad) and visualized with the Amersham 600 Imager (GE Healthcare). Images were analyzed using software ImageStudioLite (LI-COR Bioscience), and protein semi-quantitation was performed by correlating the band intensity of each sample to that of the loaded standard.

### Electroassembly of PEG-SH, Protein G-5xCys, and Antibody Interfaces

A mixture of 5 mM 1,1′-Ferrocenedimethanol (Fc) (Santa Cruz Biotechnology) and 50 mg/mL 4-arm PEG-SH (JenKam) was first prepared in phosphate buffer (0.1 M, pH 7.0). The surface of a 2 mm diameter gold standard electrode (working electrode) was fully immersed in the solution along with a platinum wire (counter electrode) and an Ag/AgCl reference electrode. PEG electrodeposition occurred for 1 min at a constant potential of 0.4 V. After PEG hydrogel formation, the surface was immersed in a solution of Fc (5 mM) and a constant voltage of 0.4 V was applied for 2 min to ensure maximal sulfenic acid group formation on the surface of the hydrogel. The protein G-5xCys functionalized surface was then generated by immersing the PEG-coated electrode in protein G-5xCys (250 μg/mL in 0.1 M PBS, pH 7.4) overnight at room temperature. After incubation, the surface was rinsed 3 times with wash buffer (0.1 M PBS, 0.05% Tween-20, pH 7.4). For the demonstration as an IgG-binding platform, the protein G-5xCys + PEG-coated electrode was incubated in 200 μL of sheep anti-rabbit IgG:DyLight^®^488 (Bio-Rad) diluted 1:1,000 in antibody buffer for 1.5 h at room temperature. To build a cell-capture platform, the protein G-5xCys+PEG-coated electrode was first incubated in 1:1,000-diluted rabbit anti-*E. coli* antibody for 2 h, followed by a 1.5-h incubation with constitutively DsRed-expressing (to facilitate visualization of cell capture) BL21 (DE3) at a cell density of OD_600_ = 1.5. Microscope images were captured with the MVX10 upright fluorescence microscope (Olympus).

## Results

### Polycysteine (5xCys) Tag Allows Protein Biofabrication on Thiolated Surfaces to Create Biohybrid Devices

**FIGURE 2:**
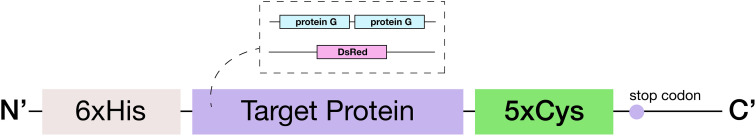
Schematic of the engineered 5xCys-tagged protein. Two target proteins: (1) red fluorescent protein DsRed and (2) two copies of *Streptococcal* protein G Fc binding domain in tandem were engineered to consist of an N-terminus 6xHis tag and a C-terminus 5xCys tag.

**FIGURE 3:**
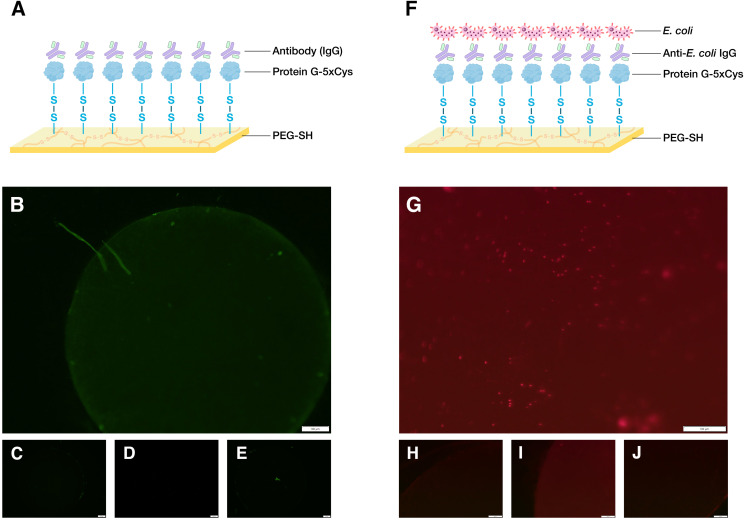
Demonstration of biodevice assemblies. **(A)** Schematic of an antibody-binding platform, image created with BioRender.com. Here, 5xCys-tagged protein G is covalently grafted onto sulfenic acid groups on the surface of electroassembled thiolated polyethylene glycol (PEG-SH). The protein G binds the Fc region of IgG. **(B)** 1.6x microscope image of a working antibody-binding platform: PEG-SH coated standard gold electrode + protein G-5xCys + IgG:DyLight 488 **(C–E)** Control images: **(C)** standard gold electrode electrodeposited with PEG-SH hydrogel **(D)** PEG-SH coated electrode + protein G-5xCys. **(E)** PEG-SH coated electrode + IgG:DyLight 488 antibody. Exposure time = 200 ms. **(F)** Schematic of a cell-capture platform. Here, the IgG captured by the protein G in **(A)** presents anti-*E. coli* antigen binding domains to the solution containing *E. coli*, enabling cell capture. **(G)** 12.6x microscope image of a working cell-capture platform: PEG-SH coated standard gold electrode + protein G-5xCys + Anti-*E. coli* IgG + *E. coli* BL21 (DE3) with constitutive DsRed expression. **(H–J)** Control Images: **(H)** standard gold electrode electrodeposited with PEG-SH hydrogel **(I)** PEG-SH coated electrode + protein G-5xCys **(J)** PEG-SH coated electrode + Anti-*E. coli* IgG + *E. coli* BL21 (DE3) with constitutive DsRed expression. Exposure time = 650 ms.

Though earlier biofabrication studies had focused on the gold-binding ability of cysteine residues (Lee et al., 2007; Bhokisham et al., 2020), in this study we sought to demonstrate its capability to form covalent bonds with thiol-containing biomaterials. To do so, we designed a polycysteine tag inserted at the C-terminus of the target protein and retained the N-terminus His-tag found in many commercial expression vectors (e.g., pET plasmids) for purification purposes (Figure 2). We had previously developed an electrodeposition method to build PEG-SH hydrogels and showed that 5xCys-tagged proteins can be oxidatively conjugated to electrodeposited hydrogels in a “targetable” manner (Li et al., 2020). Here, we further show the possibility to build multifunctional, bio-hybrid devices with such fabrication methods and 5xCys-tagged biomaterials. First, an antibody-binding platform suitable for immunoassays was built (Figure 3A) by engineering the antibody-binding *Streptococcal* protein G Fc binding domain with the 5xCys tag. Both fluorescent (Figures 3B–E) and HRP-labeled (Li et al., 2020) antibodies were shown to bind onto protein G-5xCys conjugated PEG hydrogels. Then, a cell-capture device was assembled using a similar setup (Figures 3F–J): the protein G bound to the PEG hydrogel was used to capture anti-*E. coli* antibodies. This assembly was proven to successfully capture *E. coli* from solutions of growth media over the course of an incubation period of 1–1.5 h at room temperature. Note that surfaces without the protein G-5xCys layer exposed identically to *E. coli* did not retain cells. While this is largely a result of the specificity of the anti-*E. coli* antibody, it also reflects the minimal level of non-specific binding attributed to the PEG hydrogel, consistent with many reports regarding the properties of PEGylated surfaces. Note also that the spatial selectivity attributed to this electroassembly method and the PEG surface is clearly visible at the electrode boundary in Figure 3B).

### Cellular Induction Strategies to Improve 5xCys-Tag Recombinant Expression

**FIGURE 4:**
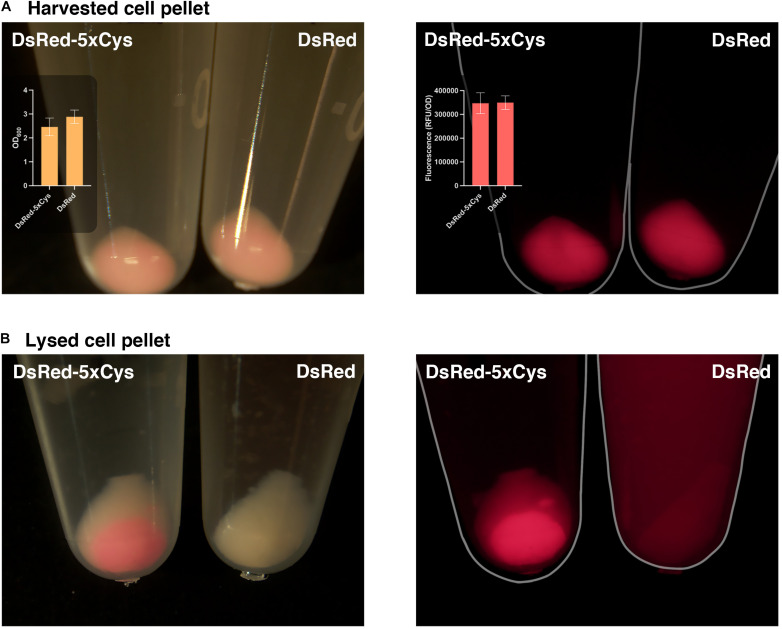
5xCys tag promotes inclusion body formation. **(A)** Harvested BL21 (DE3) cells induced with 1 mM IPTG overnight for DsRed-5xCys or DsRed expression. Left: bright view; Left inset plot: Mean OD_600_ values (*n* = 3); Right: fluorescent microscope, CY3 filter; Right inset plot: Mean red fluorescence (RFU/OD_600_) (*n* = 3). Error bars represent the standard deviation between replicates. **(B)** Cell pellets lysed with Bugbuster solution (shaking at 150 rpm for 1 h at room temperature) for soluble protein extraction. Left: bright view; Right: fluorescent microscope, CY3 filter.

During the His-tag mediated purification processing of 5xCys-tagged proteins, we had observed their yields were significantly lower when compared to the identically expressed and purified non-Cys tagged counterparts. As shown in Figure 4A, overnight expression of both DsRed and DsRed-5xCys by 1 mM IPTG-induced BL21 (DE3) appeared to be roughly equal. That is, both the pellet size and the fluorescence intensity was similar whether or not the Cys-tag was present. After a standard cell lysis procedure, however, very little soluble DsRed-5xCys was found in the protein extraction reagent and instead, the insoluble cell debris remained bright red. This was the opposite for the DsRed counterpart with no tag (Figure 4B). Hence, we hypothesized that the inserted cysteine residues promoted aggregation and thus resulted in a more prominent insoluble fraction. Presumably, the protein had agglomerated into inclusion bodies. Though active proteins could be isolated and recovered from inclusion bodies, the process was laborious and the final yield remained very low. To overcome this issue, we have attempted two strategies that focus on the host as described below.

### QS-Mediated Autoinduction Reduces Inclusion Body Formation

**FIGURE 5:**
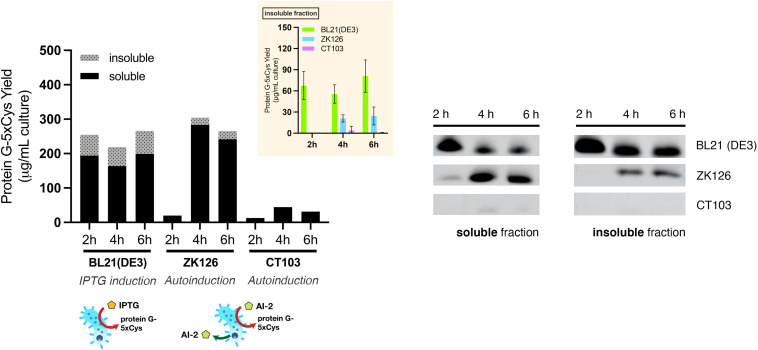
Protein G-5xCys production with conventional IPTG induction and QS-mediated autoinduction. Total yield of protein G-5xCys (μg) per mL of BL21 (DE3), ZK126 and CT103 culture, analyzed by Western blotting. Black: soluble fraction; dotted: insoluble fraction. Plotted values represent the average of biological replicates (*n* = 3). Yields of protein G-5xCys in the insoluble fractions (μg/per mL culture) are also shown in the inset plot. Error bar represents the standard deviation between replicates.

First, we sought to lessen the metabolic burden brought about by overexpression with an alternative induction method. To enable QS-mediated autoinduction, the plasmid encoding the 5xCys-tagged protein G (pET-G2Cys5) along with a “switch” plasmid (pCT5) as described in Tsao et al. (2010), were transformed into wildtype (ZK126) and an *E. coli* W3110 *lsrFG* null mutant, which is an AI-2 hypersensitive strain (CT103). This strain, lacking AI-2 processing enzymes, LsrFG, accumulates phosphorylated AI-2 in the cell cytoplasm and is denoted “hypersensitive” because the level of *lsr*-mediated transcription can be much higher than would normally be expected for a given concentration of AI-2 outside of the cell (Marques et al., 2011, 2014; Servinsky et al., 2016). The gene deletions should not alter the promoter strength, nor transcription rate, but instead, should turn on the promoter at lower AI-2 levels. In Figure 5, we show Western blot results from the autonomously induced ZK126 strain, autonomously induced CT103, and control cultures of BL21 (DE3) where IPTG is added at mid-log phase to induce the *lac* promoter. The latter represents the standard method for IPTG induction. Note that in order for the autonomously induced cells to actuate gene expression, the level of AI-2 must accumulate and subsequently be transported back into the cells where it initiates transcription via the *lsr* promoter (Figure 1). Thus, the yield in the autonomously induced ZK126 was initially low (i.e., at 2 h), but this quickly increased and eventually surpassed the yield of BL21 (DE3), a control culture with inducer IPTG added at time zero. The autoinduced culture exhibited the highest total yield and the highest soluble fraction. Interestingly, upon IPTG induction of the *E. coli* BL21 (DE3) control, the target protein seemed to accumulate rapidly, resulting in the highest amount of target protein at 2 h post-induction (Figure 5). Nonetheless, in agreement with our observations of the cell pellets, ∼ 35% of the total target protein was found in the insoluble fraction (Figure 5).

Most noteworthy was that the amount of protein G-5xCys found in the insoluble fractions of both the two autonomously induced hosts CT103 and ZK126 was significantly lower than the IPTG-induced culture. We estimated that the protein G-5xCys in the insoluble fraction of the autonomously induced ZK126 cells constituted less than 10% of the total yield. To our surprise, the yield of autonomously induced CT103 was consistently low throughout, lower than both the ZK126 autoinduced culture and the traditional IPTG-induced BL21 (DE3) expression.

These results demonstrate that even though the yield of IPTG-induced BL21 (DE3) was high, a substantial amount of the target protein was found to be insoluble and had gone to waste. With QS-mediated autoinduction, the insoluble fraction (presumably found in inclusion bodies) was shown to be drastically reduced. In the Discussion, we suggest why the CT103 results were so disappointing.

### Deletion of *oxyRS* Enhances 5xCys-Tagged Protein Production

Next, we had examined how deletion of the oxidative stress regulon, *oxyRS*, might affect the production of 5xCys-tagged proteins. Several genes that were reported to influence the cellular response to oxidative stress, such as *gor* and *ahpC*, are directly regulated by *oxyR*, and the global transcriptional activator *rpoS* is regulated by *oxyS*; while the actual disulfide-bond catalyzing *dsbB*, which is normally found in the periplasm, is not reported to be regulated by this regulon. Thus, by altering *oxyRS* functions we did not specifically target the ability or inability of cells to create disulfide bonds, rather our *oxyRS* approach deals more generally with oxidative stress, including that resulting from metabolic perturbations.

**FIGURE 6:**
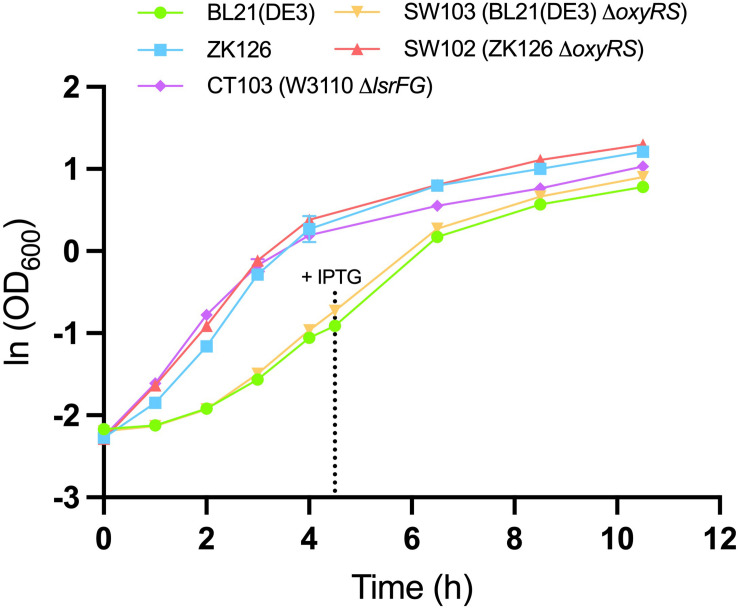
Growth of different cell cultures. Mean OD_600_ values for IPTG-induced BL21 (DE3) (green, circle), IPTG-induced SW103 (yellow, downward triangle), autoinduced ZK126 (blue, square), autoinduced CT103 (purple, diamond), and autoinduced SW102 (red, upward triangle) cultures. Both BL21 (DE3) and SW103 cultures were induced with 1mM IPTG at OD_600_ = 0.4. Error bar represents the standard deviation between replicates (*n* = 3).

**FIGURE 7:**
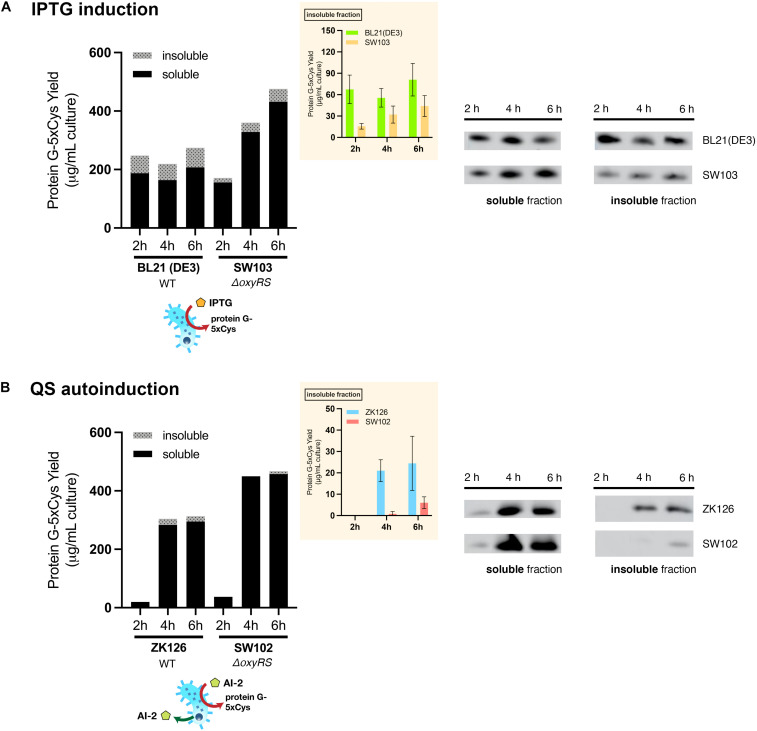
Effect of *oxyRS* deletion to protein G-5xCys expression. **(A)** Total yield of protein G-5xCys (μg) per mL of IPTG-induced BL21 (DE3) and SW103 (Δ*oxyRS*) culture analyzed by Western blotting. **(B)** Total yield of protein G-5xCys (μg) per mL of QS-autoinduced ZK126 and SW102 (Δ*oxyRS*) culture analyzed by Western blotting. Black: soluble fraction; dotted: insoluble fraction. Plotted values are the average of biological replicates (*n* = 3). Yields of protein G-5xCys in the insoluble fraction (μg/per mL culture) are also shown in the inset plot. Error bar represents the standard deviation between replicates.

To evaluate the effect of *oxyRS* deletion toward expression of 5xCys-tagged proteins, we first transformed pET-G2Cys5 into BL21 (DE3) and its *oxyRS* null mutant (SW103). We observed no significant difference in the growth rate of the *oxyRS* mutant vs. its isogenic parent (Figure 6). Interestingly, however, the Δ*oxyRS* SW103 displayed significantly higher yields in both total and soluble protein at 4- and 6-h post-induction (Figure 7A). Moreover, SW103 produced less insoluble protein (Figure 7A). These results suggest that *oxyRS* deletion alone contributes to the expression of cysteine-tagged heterologous protein in *E. coli* by both enabling its partitioning away from the insoluble fraction to the soluble fraction and by enabling more total protein.

We then tried to combine both approaches, namely the QS-mediated autoinduction and the deletion of *oxyRS*, to see if the insoluble fraction could be reduced even further. Both plasmids pCT5 and pET-G2Cys5 were transformed into ZK126’s isogenic *oxyRS* null mutant (SW102), which we created here to allow autonomously inducible expression of protein G-5xCys. Similar to ZK126 and consistent with low initial AI-2 levels, the expression of protein G-5xCys started at low levels initially (see 2 h post-induction) but increased considerably thereafter (Figure 7B). By 4 and 6 h post-induction, the total and soluble yields in autonomously induced SW102 had become higher than autonomously induced ZK126. Also, the soluble quantity of protein G-5xCys was higher and the insoluble fraction was lower in SW102 (Figure 7B). Further, both autoinduced cultures yielded less insoluble fraction of the target protein than the IPTG-induced cultures, and the two *oxyRS*-null strains displayed higher total/soluble yield compared to their wildtype counterparts. These results supported our hypothesis that both methods independently facilitate the expression of 5xCys-tagged proteins and when employed in tandem, result in even better yields. We suggest that this works by altering the metabolic and oxidative state of the host strain which, in turn, helps to maintain solubility.

## Discussion

As biohybrid devices have emerged, advances in developing tools that help to functionalize these devices will be all the more appreciated. That is, methodologies that enable integration of biological components with more traditional microdevice materials will enable significantly expanded diversity in the functions that are available. In this study, we show how a 5xCys tag that is incorporated onto the C-terminus of an IgG-binding protein G can easily and rapidly be assembled onto electrodes, preserving their function by the incorporation of simple device-born oxidation cues from the electrode. Rather than relying on electrostatic interactions (e.g., thiol-gold absorption; Di Felice et al., 2003), 5xCys tagged-proteins are capable of forming robust covalent disulfide bonds with a versatile PEG-SH hydrogel. Moreover, the PEG-SH coating, because it is in 3D, greatly increases binding sites for 5xCys tagged-protein conjugation when compared to a bare gold surface. Also, since our assembly methodology is demonstrated using a gold electrode, non-biochemical assays such as electroanalytical methods [e.g., cyclic voltammetry (CV), electrochemical impedance spectroscopy (EIS)] which allow fast and sensitive detection, can still be performed as the components are directly assembled onto the sensing electrode with the spatial resolution of the electrode (Li et al., 2020; Motabar et al., 2021). We expect many devices with more sophisticated functions can be built based on our demonstrations.

Although the cloning processes for inserting a 5xCys tag into a target protein are fairly routine, we have observed that this affinity tag presents challenges in protein expression and purification, as it seems to promote the formation of troublesome inclusion bodies. While we explored two different cellular engineering methods to overcome this problem, we made several interesting observations. First and foremost, the yield of soluble 5xCys was highest in the autoinduced host with *oxyRS* deletion. Although the conventional IPTG-induced BL21 (DE3) process delivered consistently high levels of expression upon induction, most of the protein was found in the insoluble fraction. That is, just by switching to the QS-mediated autoinduction method, we found the amount of insoluble protein had dropped significantly. This result further strengthened our previous hypothesis (Tsao et al., 2010), that QS-mediated autoinduction enables the expression host to regulate protein expression based on its metabolic state and minimizes transient product-limiting perturbations. This in contrast to manual IPTG-induction, imposing “full-on” expression regardless of how much burden it brings (Harcum and Bentley, 1993; Ramirez and Bentley, 1995; Tsao et al., 2010). This would especially benefit the expression of problematic proteins, such as the 5xCys-tagged proteins in our study that are prone to aggregate. That said, QS-mediated autoinduction required a longer expression period, as our results showed low amounts of target protein at 2 h post-induction while at the same time the expression of IPTG-induced BL21 (DE3) was high. While we have demonstrated the benefits of QS-mediated expression for a cysteine-tagged protein, benefits for rewired QS circuitry have also appeared for non-Cys-tagged proteins including GFP (Tsao et al., 2010; Zargar et al., 2016), β-galactosidase (Tsao et al., 2010), chloramphenicol acetyltransferase (CAT) and an organophosphorus hydrolase (OPH) (Tsao et al., 2011), bacterial AI-2 regulators LsrK and LsrACDB (Zargar et al., 2016). It may well be that this methodology is generally beneficial, including for proteins with designer tags.

We were also surprised to find that CT103, while having very little difference in its growth rate compared to the wildtype ZK126 (Figure 6), produced very little target protein throughout (including 2–6 h post-induction and even overnight, not shown). With the *lsrFG* deletion, we expected this strain would be extra sensitive to the autoinducer and result in higher expression since it lacks the machinery (LsrFG) to process and degrade AI-2 (Servinsky et al., 2016). There may be several factors for this observation, but more study would be required to pinpoint the main contributors: (i) the switch “on” was at a far lower OD and the transient may have resulted in the elicitation of product-degrading proteases, notably those that degrade the N-terminal His tag (we note that N-terminal degradation is common in *E. coli* (Varshavsky, 2019) (ii) the initial synthesis rate of the target protein is dependent on the first observed signal concentration (Servinsky et al., 2016) and this was likely lower in this strain in accordance with Δ*lsrFG* and a lower threshold level of AI-2 for activating *lsr* gene expression (Zargar et al., 2016). Then this trajectory of low expression was subsequently maintained throughout the expression period despite increased levels of AI-2 in later stages; and/or (iii) we have also noted that CT103, derived from W3110, contains a genomic copy of the entire *lac* operon that is deleted in ZK126 and its derivative (SW102). Since complex media such as LB broth often contains residual lactose associated with the milk protein digest, tryptone (Studier, 2014), this could influence both the *lac* operator-governed pET-G2Cys plasmid and the *lsr* operon that is glucose-repressed (Wang L. et al., 2005). As noted, additional work would help to clarify our CT103 observations.

Beyond the induction method, we also investigated whether an altered oxidative stress response contributed to this agglomeration issue, noting that the cysteine tagged protein is surely redox active. While the cytoplasm of wildtype bacteria is maintained in a reduced state, deletion of *oxyRS* will hinder its ability to eradicate H_2_O_2_, a major source of oxidative stress, and possibly shift the redox state of the cytoplasm to becoming more oxidizing (Gonzalez-Flecha and Demple, 1997, 1999). This, theoretically, will promote disulfide bond formation between two cysteine residues (Thomas and Mallis, 2001). Although it is unknown how the 5xCys tag would affect the folding of the fused protein, our results suggest that a more oxidized environment is preferred for the expression of 5xCys-tagged proteins, which helps to increase soluble target protein content and reduce inclusion body formation. By simply shutting off the oxidative stress response, perhaps this method could benefit the production of not only other Cys-tagged proteins, but other redox-sensitive proteins that require correct disulfide bond formation, such as single chain Fv antibodies (Zhang et al., 2002), antimicrobial peptide snakin-1 (Kuddus et al., 2017), and murine Wnt-1 (Mursula et al., 2006). Again, additional investigations would help us to understand the favored tertiary and quaternary structures of the 5xCys-tagged proteins, for example, how easily do they aggregate and form oligomers? Do they prefer to form inter- or intra-peptide disulfide bonds? Which conformation is more soluble while which is more likely to form inclusion bodies? Or, does the number of C-terminal cysteine residues influence these results. Answers to these questions will surely provide more insight into the phenomena observed in this study.

In summary, we have introduced a simple affinity tag to the C-terminus of a bacterial protein G, enabling its covalent tethering onto thiol-containing hydrogels. Moreover, to combat the challenging expression characteristics of this 5xCys-tagged protein, two cellular engineering methods were implemented and proved to successfully enhance its production and retain its presence in the soluble fraction. We envision these methods will benefit not only the production of the two 5xCys-tagged proteins studied here, but many other cysteine-rich proteins that are critical in biological systems.

## Data Availability Statement

The raw data supporting the conclusions of this article will be made available by the authors, without undue reservation.

## Author Contributions

SW, C-YT, and WB conceived the concepts and planned and designed the experiments. SW, C-YT, and DM performed the experiments and data analyses. SW, JL, GP, and WB wrote, discussed, and edited the manuscript. All authors contributed to the article and approved the submitted version.

## Conflict of Interest

The authors declare that the research was conducted in the absence of any commercial or financial relationships that could be construed as a potential conflict of interest.
