# Supplementary material for: A Redox-Based Autoinduction Strategy to Facilitate Expression of 5xCys-Tagged Proteins for Electrobiofabrication
Source: Front Microbiol. 2021 Jun 18;12:675729. doi: 10.3389/fmicb.2021.675729 (PMC8250426; doi:10.3389/fmicb.2021.675729)
Supplement: Supplementary file 1 [file Table_1.DOCX]

Supplementary Material

**A Redox-based Autoinduction Strategy to Facilitate Expression of 5xCys-tagged Proteins for Electrobiofabrication**

Wang *et al.*

# Supplementary Figures and Tables

## Supplementary Tables

**Supplementary Table S1.**

| **Primer Name** | **Sequence** | **Relevant Description** |
| --- | --- | --- |
| FWD_ProteinG | CACCATGGGAGTTAAGATCCGCATGAC | Upstream primer for cloning Protein G with a 5’-CACC sequence for directional cloning into pET200 |
| REV_ProteinG-Cys | CTAGCAACAACAACAACACAAGATCTTCGGGTCCATTTCCG | Downstream primer for cloning Protein G with 5xCys tag from pET-E72G3 |
| FWD_DsRed | CACCATGGCCTCCTCCGAGGAC | Upstream primer for cloning DsRed with a 5’-CACC sequence for directional cloning into pET200 |
| REV_DsRed-Cys | CTAGCAACAGCAACAACAGCAGCACACATTGATCCTAGCAGAAGCACAGG | Downstream primer for cloning DsRed with 5xCys tag from pET-DsRed |
| lsrFGHP3 | ATCGGCAAATACGATTTCTGATGTGCATTACTTAACCGGAGTAAGTTATGGTGTAGGCTGGAGCTGCTTC | Primer for deletion of gene *lsrF, lsrG* |
| lsrFGHP2 | AGATTCCAGTTTCGCGACACAGGTTTTGTAGTGGGGCGTGCATATGAATATCCTCCTTAG | Primer for deletion of gene *lsrF, lsrG* |
| oxyHP1 | CCTGTTTTAAAACTTTATCGAAATGGCCATCCATTCTTGCGCGGATGGCCGTGTAGGCTGGAGCTGCTTC | Primer for deletion of genes *oxyR, oxyS*, and the intergenic region |
| oxyHP2 | GTATAAATTTGAGCCTGGCTTATCGCCGGGCTTTTTTATGGCAAAAAAAAGCATATGAATATCCTCCTTAG | Primer for deletion of genes *oxyR, oxyS*, and the intergenic region |

(Supplementary Figure is on the following page)

## Supplementary Figures

**
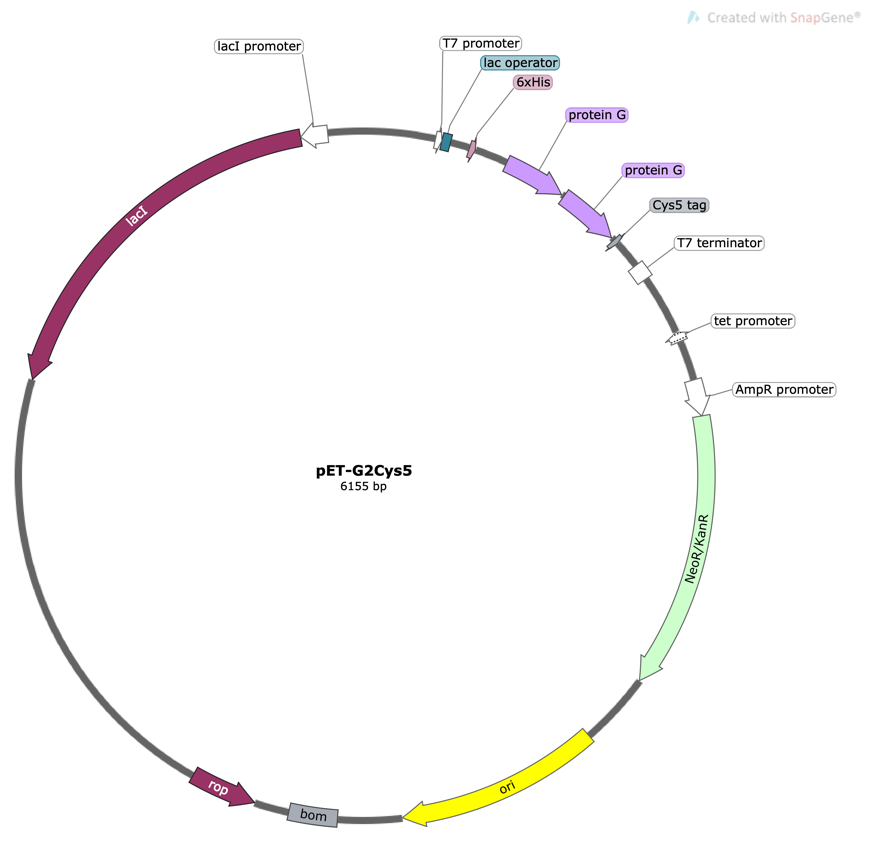
**

**Supplementary Figure S1.** **Plasmid map** **of pET-G2Cys5** The construct contains two copies of the Fc-binding domain of protein G and follows with five cysteine residues at the N-terminal.
